# Supplementary material for: Persistent representation of a prior schema in the orbitofrontal cortex facilitates learning of a conflicting schema
Source: bioRxiv. 2025 Mar 1:2025.02.28.640679. Preprint. [Version 1] doi: 10.1101/2025.02.28.640679 (PMC12190322; doi:10.1101/2025.02.28.640679)
Supplement: Supplement 1 [file NIHPP2025.02.28.640679v1-supplement-1.pdf]

bioRxiv preprint doi: https://doi.org/10.1101/2025.02.28.640679; this version posted March 1, 2025. The copyright holder for this preprint (which was not certified by peer review) is the author/funder. This article is a US Government work. It is not subject to copyright under 17 USC 105 and is also made available for use under a CC0 license.

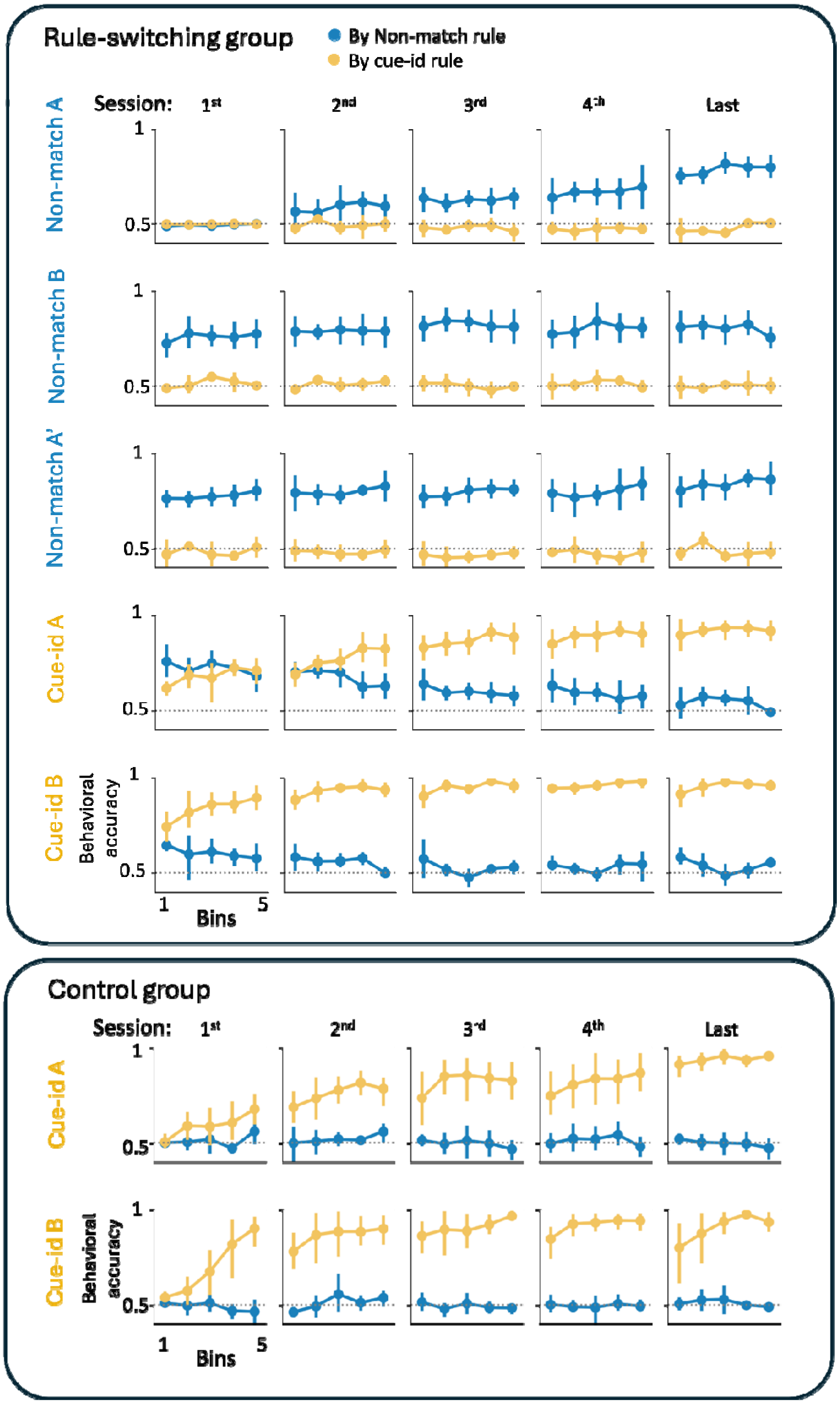

Supplementary 1. *Accuracy according to the non-match (blue) and cue-identity (yellow) rules broken down to different bins within a session.*

bioRxiv preprint doi: https://doi.org/10.1101/2025.02.28.640679; this version posted March 1, 2025. The copyright holder for this preprint (which was not certified by peer review) is the author/funder. This article is a US Government work. It is not subject to copyright under 17 USC 105 and is also made available for use under a CC0 license.

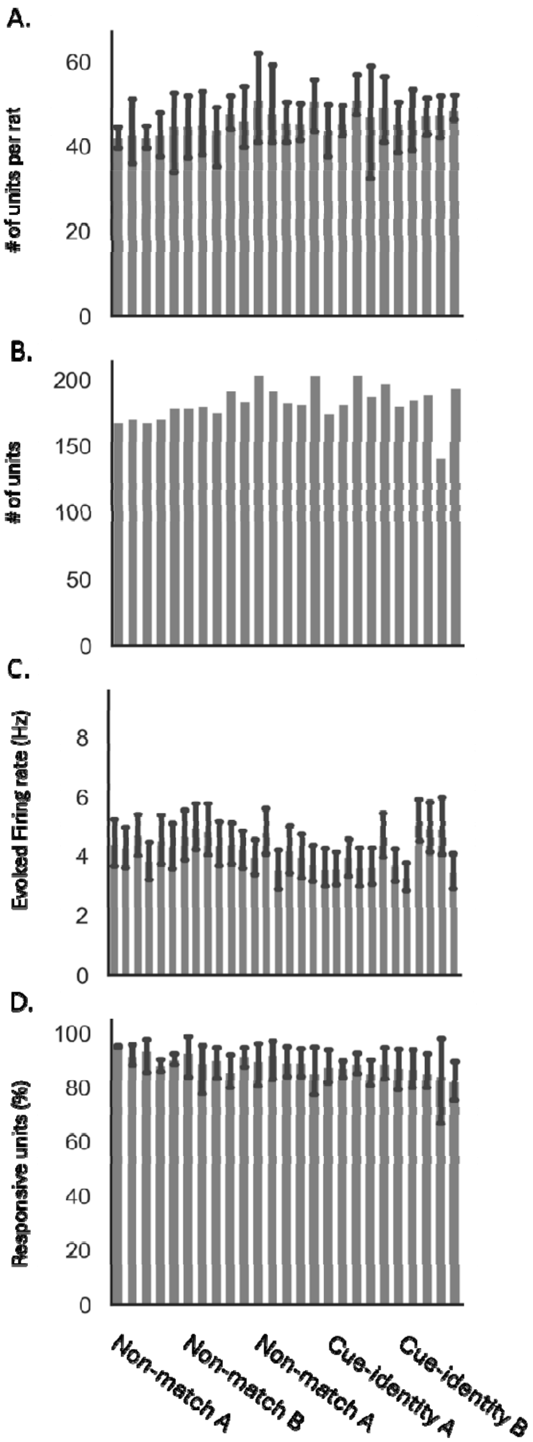

Supplementary 2. A. *single units recorded per rat per session (mean+sem).* ***B.*** *Total number of single units recorded per session.* ***C.*** *Firing rate in 500 ms preceding the decision per session (mean+sem).* ***D.*** *Percentages of units with a significant response during the odor sampling period (mean+sem).*

bioRxiv preprint doi: https://doi.org/10.1101/2025.02.28.640679; this version posted March 1, 2025. The copyright holder for this preprint (which was not certified by peer review) is the author/funder. This article is a US Government work. It is not subject to copyright under 17 USC 105 and is also made available for use under a CC0 license.

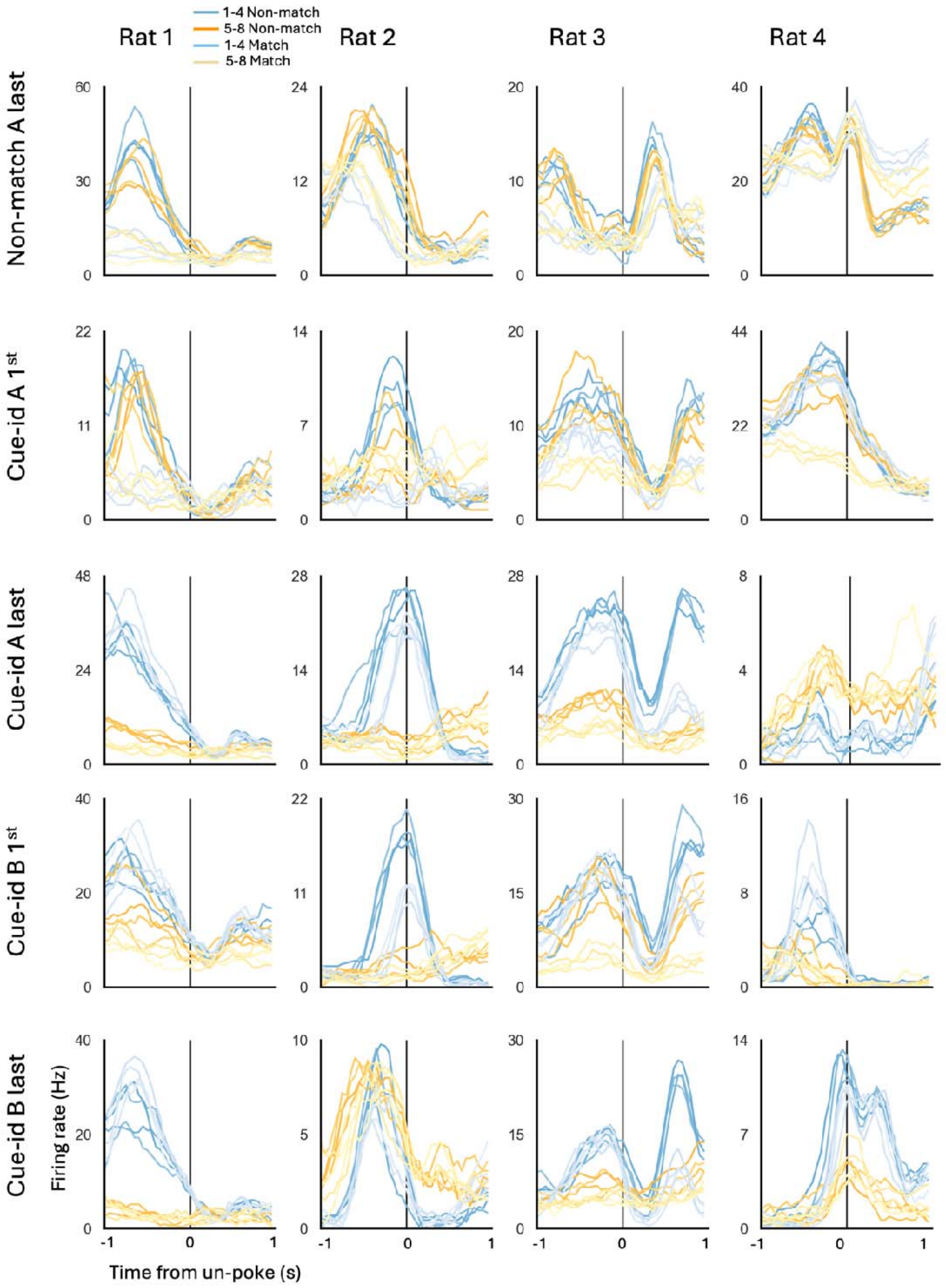

Supplementary 3. *Peristimulus time histograms (PSTHs) of example neurons, from 4 rats and 5 learning sessions, in response to 16 different trial types: 8 odors (1-4: blue lines; 5-8: yellow lines) X 2 trial configurations (match: light colors; non-match: dark colors. The PSTHs were aligned to the decision time (un-poke from odor port).*

bioRxiv preprint doi: https://doi.org/10.1101/2025.02.28.640679; this version posted March 1, 2025. The copyright holder for this preprint (which was not certified by peer review) is the author/funder. This article is a US Government work. It is not subject to copyright under 17 USC 105 and is also made available for use under a CC0 license.

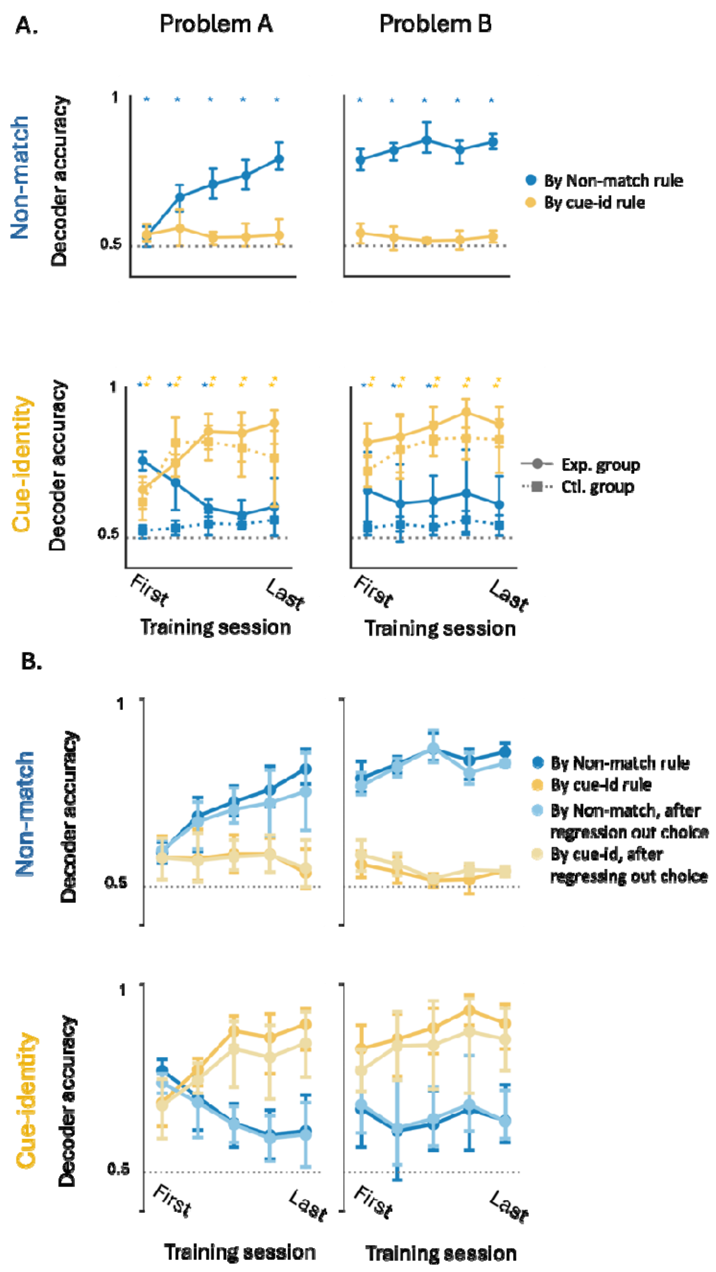

Supplementary 4. A. *Neural decoder accuracy based on the non-match rule (blue) and cue-identity rule (yellow) in the main group of rats (circles) and the control group (squares, dashed lines). Asterisks indicate statistically significant differences from shuffled data for the non-match rule (blue) and cue-identity rule (orange) (p < 0.01, permutation test). Asterisks in the upper row denote statistically significant differences for the control group.* ***B.*** *Decoder accuracy based on the non-match rule (blue) and cue-identity rule (yellow), shown before (dark lines) and after (light lines) regressing out the rats' choice (go/no-go).*
